# Genetically Predicted Plasma Cortisol and Common Chronic Diseases: A Mendelian Randomization Study

**DOI:** 10.1111/cen.14966

**Published:** 2023-09-05

**Authors:** Wei-Hsuan Lee, Susanna C. Larsson, Angela Wood, Emanuele Di Angelantonio, Adam S. Butterworth, Stephen Burgess, Elias Allara

**Affiliations:** 1 British Heart Foundation Cardiovascular Epidemiology Uni, Department of Public Health and Primary Care, University of Cambridge, Cambridge, UK; 2 Unit of Cardiovascular and Nutritional Epidemiology, Institute of Environmental Medicine, Karolinska Institutet, Stockholm, Sweden; 3 Unit of Medical Epidemiology, Department of Surgical Sciences, Uppsala University, Uppsala, Sweden; 4 Victor Philip Dahdaleh National Institute for Health Research Blood and Transplant Research Unit in Donor Health and Genomics, University of Cambridge, Cambridge, UK; 5 British Heart Foundation Centre of Research Excellence, University of Cambridge, Cambridge, UK; 6 Health Data Research UK Cambridge, Wellcome Genome Campus and University of Cambridge, Cambridge, Cambridge, UK; 7 Cambridge Centre of Artificial Intelligence in Medicine, Cambridge, UK; 8 Health Data Science Research Centre, Human Technopole, Milan, Italy; 9 MRC Biostatistics Unit, University of Cambridge, Cambridge, UK

**Keywords:** cortisol, Mendelian randomization, chronic diseases, type 2 diabetes, hypertension, osteoporosis, depression, schizophrenia.

## Abstract

**Objective:**

Cushing’s syndrome is characterized by hypercortisolaemia and is frequently accompanied by comorbidities such as type 2 diabetes, hypertension, osteoporosis, depression and schizophrenia. It is unclear whether moderate but lifelong hypercortisolaemia is causally associated with these diseases in the general population. We aimed to address this research gap using a Mendelian randomization approach.

**Methods:**

We used three cortisol-associated genetic variants in the *SERPINA6/SERPINA1* region as genetic instruments in a two-sample, inverse-variance weighted Mendelian randomization analysis. We obtained summary-level statistics for cortisol and disease outcomes from publicly available genetic consortia, and meta-analysed them as appropriate. We conducted multivariable Mendelian randomization analysis to assess potential mediating effects.

**Results:**

A one standard deviation higher genetically predicted plasma cortisol was associated with greater odds of hypertension (odds ratio 1.12, 95% confidence interval [CI] 1.05-1.18) as well as higher systolic blood pressure (mean difference [MD] 0.03 SD change, 95% CI 0.01-0.05) and diastolic blood pressure (MD 0.03 SD change, 95% CI 0.01-0.04). There was no evidence of association with type 2 diabetes, osteoporosis, depression and schizophrenia. The association with hypertension was attenuated upon adjustment for waist circumference, suggesting potential mediation through central obesity.

**Conclusion:**

There is strong evidence for a causal association between plasma cortisol and greater risk for hypertension, potentially mediated by obesity.

## Introduction

Cortisol plays a crucial role in maintaining metabolic homeostasis in the human body. While cortisol variations in the normal range are essential to the physiologic response to internal and external stressors, abnormal cortisolaemia is a presenting feature of Cushing’s Syndrome (CS), an endocrinologic chronic condition. In more than half of CS patients, CS is accompanied by a range of comorbidities, such as type 2 diabetes,^1–3^ hypertension,^1–4^ osteoporosis,^2–5^ and neuropsychiatric disorders.^2,4^

The role of hypercortisolaemia in the aetiology of these chronic diseases in the general population is, however, less clear. Observational evidence shows that persistent hypercortisolism is associated with higher risk of cardiometabolic disorders such as abdominal obesity,^6–8^ type 2 diabetes,^9^ and hypertension.^6–10^ Neuropsychiatric patients also show cortisol dysfunction.^11^ While previous studies report associations between cortisol and chronic diseases in non-CS patients, the interpretation of these findings is limited by the intrinsic limitations of observational evidence, such as potential reverse causality and residual confounding.

Additionally, evidence from observational studies suggests that exposure to major environmental stressors is associated with these diseases in the general population. For example, exposure to major earthquakes was associated with raised glycated haemoglobin and higher blood pressure, but also with lower risk of suicide.^12^ However, the mechanisms by which this occurs is often unknown. Because cortisol is secreted in the presence of internal and external stressors,^13^ elucidating causal associations between hypercortisolaemia and common chronic diseases may provide insight into the mechanistic link that mediates the effects of chronic stress on health in the general population.

We used a Mendelian randomization (MR) approach to investigate potential associations of genetically predicted cortisol with common chronic diseases. We selected five chronic diseases that are frequently comorbid in CS patients,^1,3,14–17^ and for which genetic data are publicly available: type 2 diabetes, hypertension, osteoporosis, depression, and schizophrenia. By performing this MR study, we aimed to provide information on the long-term effects of cortisol on the onset of common chronic diseases in the general population.

## Methods

### Study design

We used a two-sample MR design, where summary statistics for the genetic associations with plasma cortisol were obtained from the CORtisol NETwork (CORNET) consortium^18^ and genetic associations with the five chronic diseases were obtained from publicly available summary statistics from genetic consortia (Figure S1).^19–24^ For statistically significant cortisol-disease associations in the main univariable analysis, we further explored associations with related traits. As some of the included diseases have different prevalence in men and women, we also conducted a sex-specific subgroup analysis in the UK Biobank (UKB) cohort.

### Genetic instrument for cortisol

The genetic instruments selected as predictors for plasma cortisol are three single nucleotide polymorphisms (SNPs) (rs2749527, rs12589136 and rs11621961) that reside in the *SERPINA6/SERPINA1* gene region and are strongly associated with plasma cortisol (*P*-value 4.0×10^-8^ to 3.3×10^-12^) in a previous genome-wide association study (GWAS) of total plasma cortisol sampled in the morning.^18^ *SERPINA6* and *SERPINA1* encode corticosteroid binding globulin (CBG) and α1-antitrypsin, respectively. CBG is a key regulator of cortisol delivery to target tissues and inflammatory sites. α1-antitrypsin is an important player in the acute phase reaction during cellular stress and inflammation, and its deficiency has been associated with CBG cleavage,^25^ thus affecting cortisol homeostasis. Further, both *SERPINA6* and *SERPINA1* are highly expressed in cortisol-relevant sensitive tissues, such as the liver and pancreas (Figure S2). These three SNPs are in low linkage disequilibrium (R^2^ 0.074-0.265 in European populations) and together explain 0.54% of the variation in morning plasma cortisol level. They have been used in several previous MR studies to investigate the role of cortisol in cardiovascular risk,^26^ atrial fibrillation,^27^ venous thromboembolism^28^ and cancer.^29^ It is worth noting that genetic instruments with similar or lower variance explained (0.1-0.4%) have been used in previous MR studies assessing associations of circulating lipids with cardiovascular outcomes^30^ and of alcohol drinking with cardiovascular disease and cancer.^31,32^ Taken together, GWAS evidence, biological plausibility, gene expression data and previous investigations indicate that the selected SNPs are likely to be valid genetic instruments for plasma cortisol levels.

### Data sources

We acquired summary statistics for cortisol-associated SNPs and targeted outcomes from GWAS meta-analyses and genetic consortia as shown in Table 1. In UKB, we performed individual-participant analyses, and case definitions for UKB participants are outlined in Table S1. The same table also includes outcome definitions for all the studies (and their participating cohorts) from which we obtained summary statistics. All studies included individuals of European ancestries. Informed consent and ethical approval were previously obtained from participants in included studies.^18–24^ We pooled together datasets that had minimal (<2%) participant overlap, while presenting also estimates from individual studies in all results tables and plots. Associations between the three plasma cortisol-associated SNPs and their associations with the five main disease outcomes and two related traits are shown in Table S2.

### Power calculation

We conducted power calculations assuming a 0.05 significance level and 0.54% variance explained by the genetic instrument.^33^ There are challenges assuming a different true OR and regression coefficient given differing demographics in study population, outcome assessment methods, and other characteristics of the study design among observational studies. Hence, we used the most conservative estimate (OR=1.18 per SD change of cortisol levels) available in a previous study^34^ to approximate the potential impact of plasma cortisol on binary outcomes and minimize the impact of between-study heterogeneity and bias. Results show power greater than 70% in pooled analyses for most diseases included in this study (Table S3).

### Statistical analysis

Prior to performing MR analysis, we meta-analysed genetic estimates from the datasets included in our study (Table S2) using a fixed effect model. We then conducted separate MR analyses for each outcome of interest using a fixed-effect inverse-variance weighted method. We accounted for the between-variant correlation matrix using data from the 1000 Genomes Project (Table S4). We generated beta-beta scatter plots of exposure-outcome associations to visually assess consistency between individual genetic associations and MR results. To assess potential pleiotropy, we performed sensitivity analysis of the main associations using MR Egger regression. To investigate the possible mediating effect of waist circumference, which is strongly related to CS, on hypertension, we conducted multivariable MR analysis. We also performed sex-specific analyses using individual-participant data in UKB. To account for false-positive inflation due to multiple hypothesis testing, we utilized a Bonferroni-corrected *P*-value lower than 0.01 (0.05/5) as a significance threshold, and we defined associations with 0.01 ≤ *P* < 0.05 as “nominal”. We performed all MR analyses in R version 3.6.0 with the MendelianRandomization package version 0.5.1.

## Results

**Figure 1:**
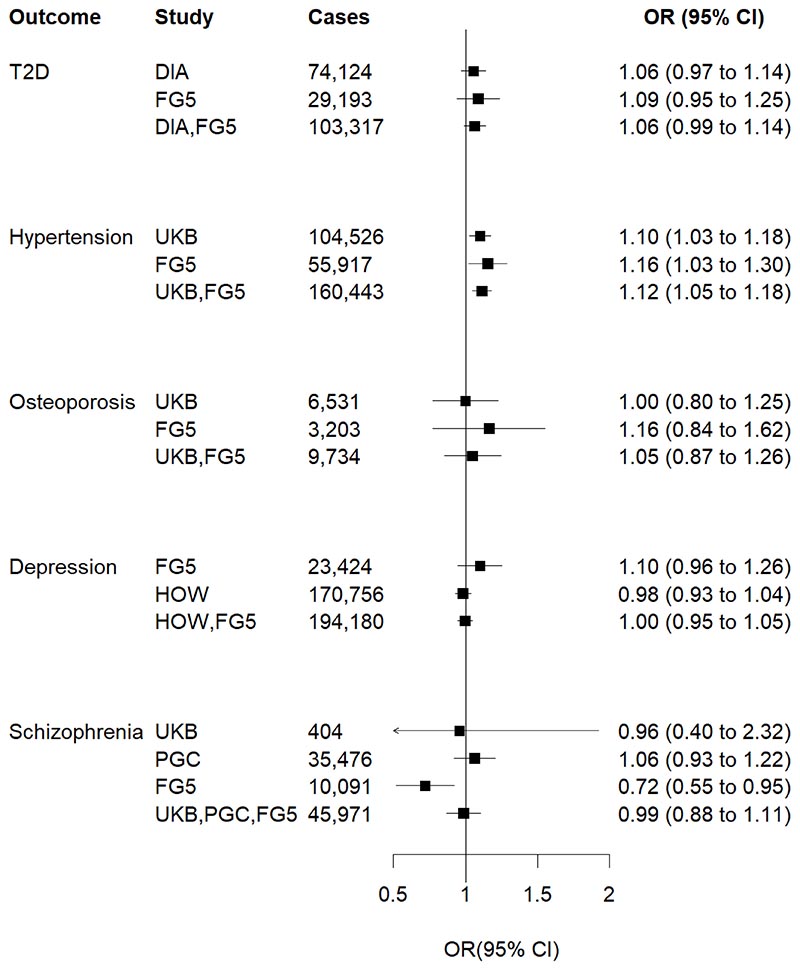
Associations between genetically predicted plasma cortisol and five main binary outcomes Estimates were scaled per one standard deviation increase in plasma cortisol levels and were derived from a univariable fixed effects inverse-variance weighted Mendelian Randomization analysis. Pooled analyses show a strong association with greater risk of hypertension (*P*=2.77×10^-4^) that was consistent in all individual studies. OR, odds ratio; T2D, type 2 diabetes; DIA, DIAGRAM Consortium; UKB, UK Biobank; FG5, FinnGen Freeze 5 consortium; HOW, Howard 2019 (see references); PGC, Psychiatric Genomics Consortium.

**Figure 2:**
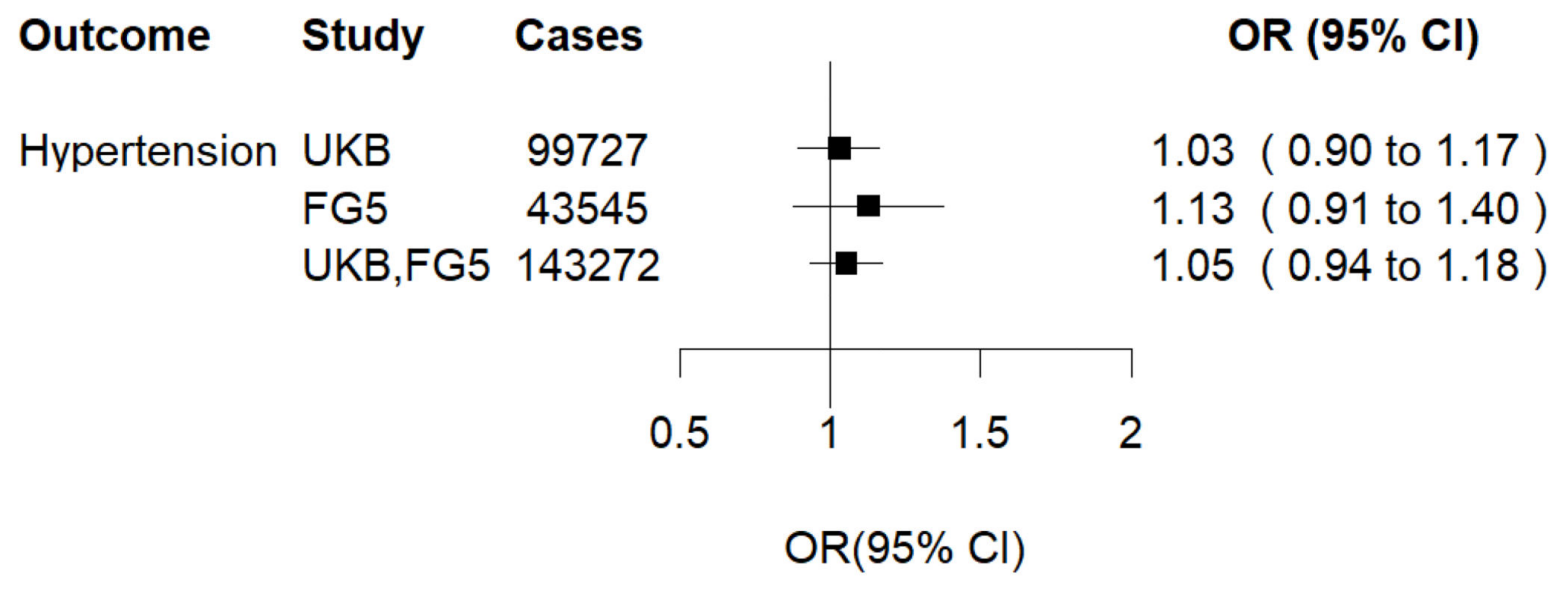
Multivariable MR on hypertension adjusting for waist circumference Effect estimates attenuated to the null in all datasets as well as in the pooled estimate. OR, odds ratio; UKB, UK Biobank; FG5, FinnGen Freeze 5 Consortium.

**Figure 3:**
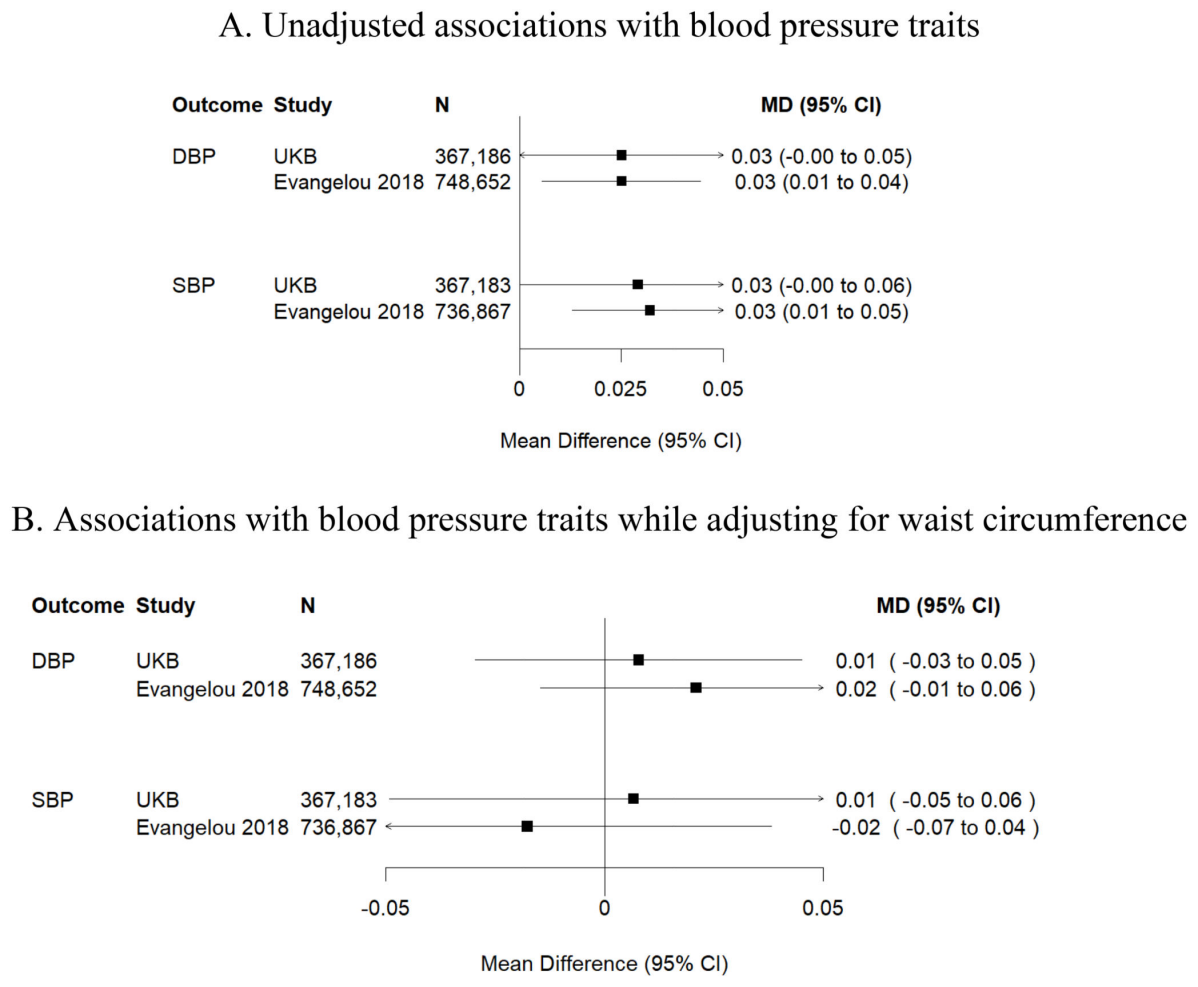
Associations of genetically predicted plasma cortisol with blood pressure traits Figure 3A. Unadjusted associations between genetically predicted plasma cortisol and two continuous traits related to hypertension. Estimates were scaled per one standard deviation increase in plasma cortisol levels. Results based on publicly available data from the Evangelou 2018 cohort (see references), which includes ~66% UKB participants, showed cortisol associations with DBP (*P*=0.023) and SBP (*P*=3.56×10^-3^). Figure 3B. These estimates were derived from a multivariable MR analysis adjusting for waist circumference. This analysis shows that associations of genetically predicted cortisol with blood pressure traits do not persist after waist circumference adjustment. UKB, UK Biobank; DBP, diastolic blood pressure; SBP, systolic blood pressure.

One standard deviation (SD) higher genetically predicted plasma cortisol was associated with 12% higher risk of hypertension (odds ratio [OR] 1.12, 95% confidence interval [CI] 1.05-1.18, *P*=2.77×10^-4^) (Figure 1). The association with hypertension did not persist in the multivariable MR analysis adjusting for genetically predicted waist circumference (Figure 2), suggesting potential mediation through central obesity. Genetically predicted cortisol was associated with both systolic blood pressure (mean difference per one-SD higher cortisol [MD] 0.03 SD change, 95% CI 0.01-0.05, *P*=3.56×10^-3^) and, nominally, with diastolic blood pressure (MD 0.03 SD change, 95% CI 0.01-0.04, *P*=0.023) (Figure 3A). These associations were much attenuated after waist circumference adjustment (Figure 3B), consistent with the multivariable analyses for binary hypertension. In sex-specific univariable MR analyses in UKB, overlapping confidence intervals suggest no evidence for differences between men (OR 1.14, 95%CI 1.04-1.26) and women (OR 1.06, 95% CI 0.96-1.17).

Beta-beta scatter plots (Figure S3) show that individual genetic associations with cortisol and each outcome are consistent with the findings of the MR analysis presented above. Sensitivity analyses using MR Egger regression showed no evidence of pleiotropy for hypertension (MR Egger intercept p=0.52), systolic blood pressure (p=0.83) and diastolic blood pressure (p=0.93).

## Discussion

This MR study utilizes genetic variants strongly associated with cortisol to examine its causal association with a range of common chronic outcomes: type 2 diabetes, hypertension, osteoporosis, depression, and schizophrenia. Our analytical approach minimizes confounding from environmental exposures and the effects of reverse causality, that are common limitations in observational studies. Our results showed that higher genetically predicted plasma cortisol is associated with a higher risk of hypertension after accounting for false-positive inflation due to multiple-hypothesis testing. An association with higher systolic blood pressure was also found, further corroborating this finding. No evidence of association was found with type 2 diabetes, osteoporosis, depression and schizophrenia.

Interestingly, associations with hypertension and blood pressure traits diminished upon waist circumference adjustment, suggesting that these associations may be driven by central obesity. This aligns with studies that have shown strong associations between increased long-term cortisol levels, measured in scalp hair, and central obesity,^35,36^ and observational associations between central obesity and hypertension.^37,38^ The biological basis of this mediation appears plausible. Cortisol changes the amount of intra-abdominal and intra-vascular fat, resulting in altered endothelial function,^39^ which in turn promotes release of tissue-derived angiotensinogen in circulation and peripheral vasoconstriction.^40^

It is worth noting that the null findings regarding the associations of cortisol with type 2 diabetes, osteoporosis and neuropsychiatric diseases should not be interpreted as conclusive evidence that cortisol does not play a causal role in the aetiology of these diseases. More powerful genetic instruments and larger outcome datasets may be able to capture weaker associations with these diseases.

There are a few limitations to our study. First, the genetic instruments explained 0.54% of the variance in morning plasma cortisol, which may render the analysis underpowered for some associations. However, power calculations show reasonable statistical power for most outcomes under the assumption of an approximately 20% risk increase. Second, it is possible that cortisol-associated variants in the *SERPINA6/SERPINA1* region may have pleiotropic effects. There is, however, clear biological plausibility for this region, which limits the possibility of horizontal pleiotropy, and the genes in this region are highly expressed in cortisol-relevant tissues such as the liver and pancreas. Additionally, MR Egger analysis showed no evidence of horizontal pleiotropy in relation to hypertension and blood pressure traits, although this may be limited by potential power issues. While we cannot completely rule out horizontal pleiotropy with the information available to date, is seems unlikely based on a combination of biological plausibility, expression data and statistical analysis. Third, our datasets include only European-ancestry cohorts which limits generalizability of study findings to other populations. Fourth, while a strength of this paper is inclusion of the main publicly available information for the outcomes analysed, their definitions may change slightly in different studies, which means that some level of heterogeneity is inevitable. We noted, however, that the associations with hypertension and blood traits were consistent in all the datasets analysed, suggesting that heterogeneity is unlikely to affect the main results of this study.

In summary, the findings of this study highlight the potential relevance of lifelong and moderate hypercortisolaemia to hypertension in general populations. In general practice, non-CS patients with hypertension and obesity not explained by conventional risk factors may need investigation for potential hypercortisolaemia. In general populations, large-scale longitudinal studies might be able to clarify whether cortisol contributes to mediating the effects of major environmental stressors on hypertension.

## Supplementary Material

Supplementary Material

## Figures and Tables

**Table 1:** Study Overview: Sample size and pooled GWAS for each outcome of interest

Main Outcomes	Pooled Consortia	N	Cases
T2D	DIA, FG5	1,109,896	103,317
Hypertension	UKB, FG5	586,296	160,443
Osteoporosis	UKB, FG5	580,320	9,734
Depression	HOW, FG5	715,843	194,180
Schizophrenia	UKB, FG5, PGC	668,649	45,971
**Associated Traits**	**Pooled Consortia**	**N**	
SBP	Evangelou 2018	736,867	
DBP	Evangelou 2018	748,652	

UKB, UK Biobank; FG5, FinnGen Freeze 5 consortium; PGC, Pyschiatric Genomics Consortium; HOW, Howard 2019; DIA, DIAGRAM Consortium; T2D, type 2 diabetes; SBP, systolic blood pressure; DBP, diastolic blood pressure.

## Data Availability

The data needed to replicate our findings are available in Table S2.
