# Supplementary material for: Genetically Predicted Plasma Cortisol and Common Chronic Diseases: A Mendelian Randomization Study
Source: Clin Endocrinol (Oxf). Author manuscript; Available in PMC 2024 Mar 1. (PMC7615603; doi:10.1111/cen.14966)
Supplement: Supplementary Material [file EMS190938-supplement-Supplementary_Material.pdf]

Figure S1. Conceptual framework of examining cortisol and chronic disease association with MR. Under MR, cortisol-associated genetic variants serve as unmodifiable proxy to the modifiable cortisol risk factor. If variants are not associated with confounders (known and unknown), they can allow for investigation of a theoretically unconfounded causal association between plasma cortisol levels and the five disease outcomes of interest. Such causal association is only valid when instrumental variable (IV) assumptions are satisfied, including relevance (IV1), exchangeability (IV2), and exclusion restriction (IV3). **Figure adapted from Lawlor 2008.**[1]

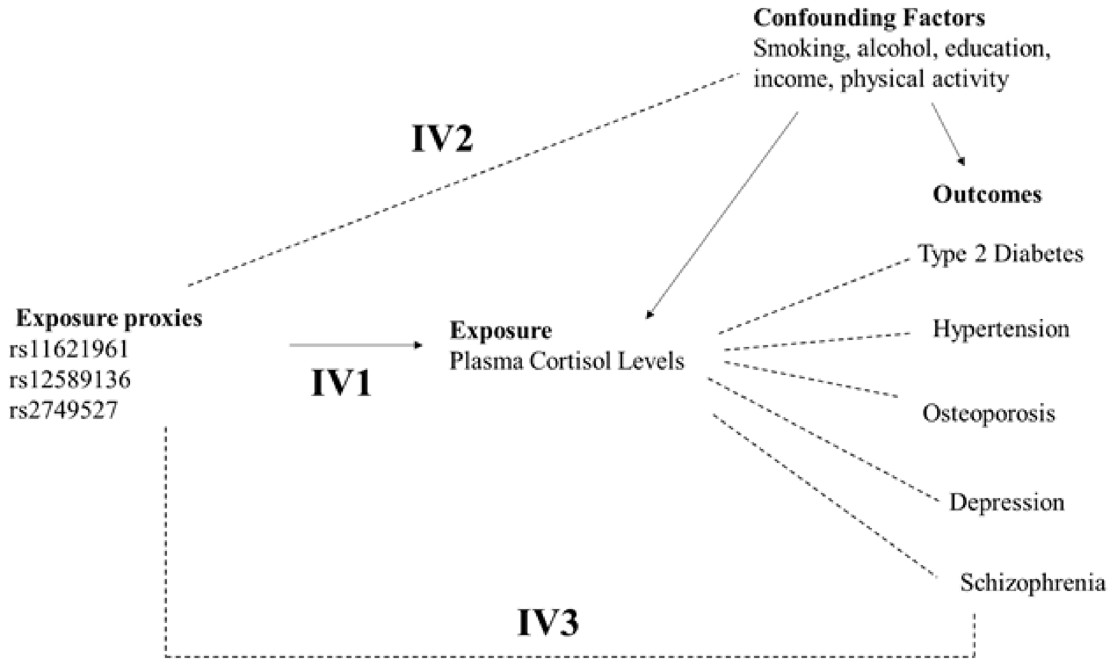

Figure S2. Log-transformed gene expression level of SERPINA6 (A) and SERPINA1 (B) in different tissue types. (A). Gene expression is highest in the liver, followed by pancreas and kidney, which are cortisol-relevant and cortisol-sensitive tissues. (B) Gene expression is highest in liver and whole blood. Graph generated in and extracted from GTExPortal.

A

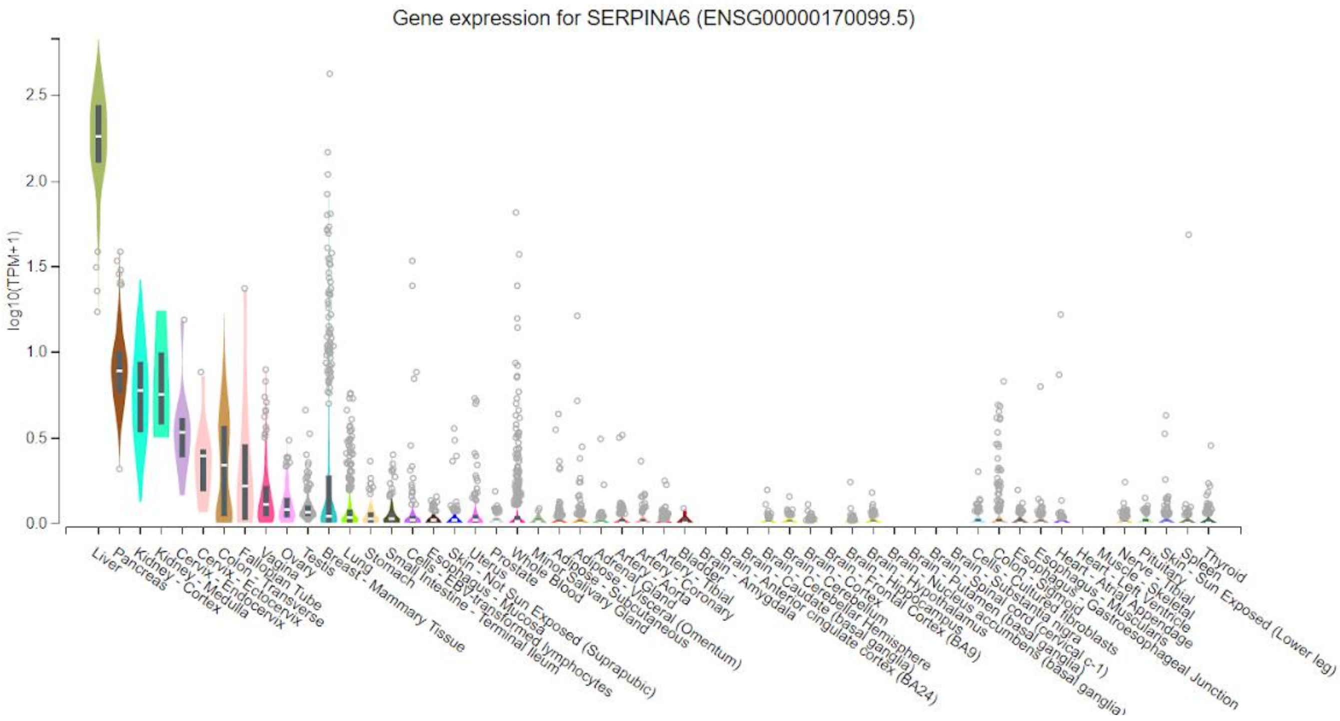

B

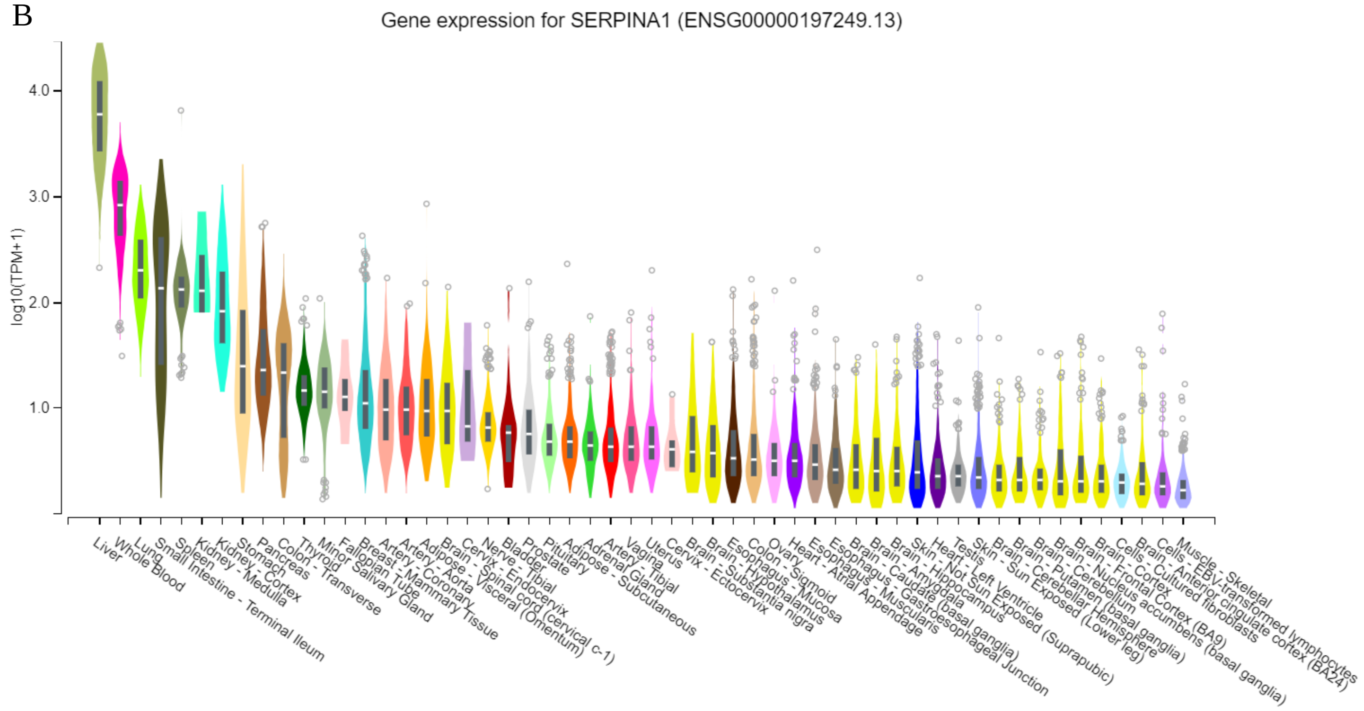

Table S1. Outcome definitions.

Outcome	Study	Subcohort	Definitions
T2D	DIA	BioME	T2D (Status as defined by algorithm)
		deCODE	T2D diagnosis is based on diagnostic fasting glucose or HbA1c levels, hospital discharge diagnosis, use of oral diabetes medication or self report.
		DGDG	T2D was diagnosed in hospital using medical history of hba1c and fasting glucose
		DGI	Patients with T2D were classified according to WHO (1999) criteria with fasting plasma glucose ≥ 7.0 mmol/l or a 2 hr glucose ≥ 11.1 mmol/l during an oral glucose tolerance test (OGTT). To avoid admixture with type 1 diabetes, patients had an age at onset > 35 years and no detectable glutamic acid decarboxylase antibodies (GAD Ab). Members from families with carriers of mutations causing maturity onset diabetes of the young (MODY; HNF4A, GCK, TCF1, IPF1, TCF2) were excluded, except for Skara where no screening for MODY mutations had been performed.
		EGCUT_ExomeCore	Previous T2D diagnosis
		EGCUT_Human370 CNV	Previous T2D diagnosis
		EGCUT_OmniExpre ss	Previous T2D diagnosis
		FHS	On treatment or fasting glucose ≥ 7 (when available) or casual glucose ≥ 11.1
		FUSION	WHO 1999 criteria of FG>=7.0 mmol/l or 2-hour plasma glucose >= 11.1 mmol/l or reported diabetes medication use or based on medical record review; no know or probable type 1 diabetes among first degree relatives; excluded if insulin treatment inititated within 10 years of disease diagnosis, detectable levels of anti-GAD antibodies and fasting C-peptide <= 0.30 nmol/l; excluded if insulin treatment inititiated within 4 years of diagnosis and fasting C-peptide <= 0.30 nmol/l
		GCKD	antidiabetic medication (ATC code A10*) or HbA1c >= 6.5%
		GENOA	Use of T2D medications or fasting glucose ≥ 7.0 mmol/l.
		GERA	ICD-9 codes in linked electronic medical health records
		GoDARTS	Patients with T2D were identified from electronic medical records [PMCID:2127363].
		GOMAP-TEENAGE	Previous diagnosis of T2D with or without psychiatric disease
		HPFS	Type 2 diabetes cases were defined as self-reported diabetes confirmed by a validated supplementary questionnaire. For cases before 1998, we used the National Diabetes Data Group criteria to define type 2 diabetes. We used the American Diabetes Association diagnostic criteria for type 2 diabetes diagnosis from 1998 onward.
		INTERACT_coreexo me	All individuals in InterAct were free of known diabetes at baseline. Ascertainment of incident T2D involved a review of existing EPIC datasets at each centre using multiple sources of evidence including self-report (self reported history of T2D, doctor diagnosed T2D, diabetes drug use), linkage to primary care registers, secondary care registers, medication use (drug registers), hospital admissions and mortality data. Information from any follow-up visit or external evidence with a date later than the baseline visit was used. Cases in Denmark and Sweden were not ascertained by self-report, but identified via local and national diabetes and pharmaceutical registers and hence all ascertained cases were considered to be verified. For centres other than those from Denmark and Sweden, we sought further evidence for all cases with information on incident T2D from no fewer than 2 independent sources, including individual medical records review in some centres.
		INTERACT_GWAS	All individuals in InterAct were free of known diabetes at baseline. Ascertainment of incident T2D involved a review of existing EPIC datasets at each centre using multiple sources of evidence including self-report (self reported history of T2D, doctor diagnosed T2D, diabetes drug use), linkage to primary care registers, secondary care registers, medication use (drug registers), hospital admissions and mortality data. Information from any follow-up visit or external evidence with a date later than the baseline visit was used. Cases in Denmark and Sweden were not ascertained by self-report, but identified via local and national diabetes and pharmaceutical registers and hence all ascertained cases were considered to be verified. For centres other than those from Denmark and Sweden, we sought further evidence for all cases with information on incident T2D from no fewer than 2 independent sources, including individual medical records review in some centres.
		KORA	T2D was self reported
		MESA	Known diabetes or fasting whole-blood glucose >7 mmol/l

Outcome	Study	Subcohort	Definitions
		METSIM	WHO 1999 criteria of FG>=7.0 mmol/l or 2-hour plasma glucose >= 11.1 mmol/l or reported diabetes medication use or based on medical record review; no know or probable type 1 diabetes among first degree relatives; excluded if insulin treatment inititated within 10 years of disease diagnosis, detectable levels of anti-GAD antibodies and fasting C-peptide <= 0.30 nmol/l; excluded if insulin treatment inititiated within 4 years of diagnosis and fasting C-peptide <= 0.30 nmol/l
		MGI	EHR-derived ICD-9 codes: 250.00,250.02,250.20,250.22,250.30,250.32,250.80,250.82,250.90,250.92
		NHS	Type 2 diabetes cases were defined as self-reported diabetes confirmed by a validated supplementary questionnaire. For cases before 1998, we used the National Diabetes Data Group criteria to define type 2 diabetes. We used the American Diabetes Association diagnostic criteria for type 2 diabetes diagnosis from 1998 onward.
		NUGENE	Include patients with Type 2 Diabetes diagnosis based on ICD9 codes (excluding those with ketoacidosis codes); then Exclude patients (currently) treated only with insulin AND have never been on a type 2 diabetes medication, and: diagnosed with T1DM, or even if not diagnosed with T1DM, diagnosed with T2DM on < 2 dates in an encounter or problem list. Also include patients with hemoglobin A1C lab value ≥ 6.5%, fasting glucose > 125 mg/dl or random glucose > 200 mg/dl AND prescribed T2D medication.
		PIVUS	Known T2D or fasting whole blood glucose > 6.1
		PROSPER	T2D was defined as having known diabetes Mellitus or fasting blood glucose >7 mmol/L
		RS1	T2D were ascertained through active follow-up using general practitioners' records, glucose hospital discharge letters and glucose measurements from the Rotterdam Study visits. According to the WHO guidelines, type 2 diabetes was defined as a fasting blood glucose >7.0 mmol/L, or the use of blood-glucose-lowering medication. Information regarding the use of blood-glucose lowering medication was derived from both structuredhome interviews and linkage to pharmacy records.
		RS2	T2D were ascertained through active follow-up using general practitioners' records, glucose hospital discharge letters and glucose measurements from the Rotterdam Study visits. According to the WHO guidelines, type 2 diabetes was defined as a fasting blood glucose >7.0 mmol/L, or the use of blood-glucose-lowering medication. Information regarding the use of blood-glucose lowering medication was derived from both structuredhome interviews and linkage to pharmacy records.
		RS3	T2D were ascertained through active follow-up using general practitioners' records, glucose hospital discharge letters and glucose measurements from the Rotterdam Study visits. According to the WHO guidelines, type 2 diabetes was defined as a fasting blood glucose >7.0 mmol/L, or the use of blood-glucose-lowering medication. Information regarding the use of blood-glucose lowering medication was derived from both structuredhome interviews and linkage to pharmacy records.
		UK BioBank	Prevalent T2D status was defined using self-reported medical history and medication in UK Biobank participants
		ULSAM	Hospital discharge register-defined diabetes before 2002
		UPCH	T2D was defined based on self-report, anti-diabetic treatment, fasting plasma glucose >7.0 mmol/L or 2-hr plasma glucose >11.1 mmol/L
		WTCCC	T2D was defined as current prescribed treatment with sulphonylureas, biguanides, other oral agents and/or insulin or in the case of individuals treated with diet alone, historical or contemporary laboratory evidence of hyperglycemia
	FG5	-	ICD9: 250.A. ICD10: E11.
Hypertension	UKB	-	ICD9: 401. ICD10: I10. Self-report (field 20002): 1065 1072.
	FG5	-	ICD8: 400, 401, 402, 403, 404. ICD9: 4019X, 4029A, 4029B, 4039A, 4040A, 4059A, 4059B, 4372A, 4059X. ICD10: I10, I11, I12, I13, I15, I674.
Osteoporosis	UKB	-	ICD9: 7330. ICD10: M80, M81. Self-report (field 20002): 1309.
	FG5	-	ICD8: 7230, 72391. ICD9: 733[0-1]. ICD10: M8[0-2].
Depression	HOW	23andMe	Subjects with depression were identified through self-report in web-based surveys. A total of six survey data sources were used to compose the depression phenotype. More information available in PMID: 27479909.
		BOMA 1-3	DSM-IV MDD; German ancestry; ≥18yo
		CoFaMS 4	DSM-IV MDD
		PsyCoLaus 5	DSM-IV MDD, age 35-66
		Edinburgh 6	DSM-IV MDD
		GenRED1 7,8	DSM-IV rMDD (recurrent or >3 y duration) & onset <31 y; FHx MDD in sib or parent
		NEWMEDS-GENPOD 9,10	ICD-10 MDD
		DGN 11	DSM-IV MDD (recurrent)
		GenRED2 7	DSM-IV MDD recurrent (or episode lasting >3y) & age onset <31y; FHx MDD in sibling or parent
		GSK/MPIP 12	DSM-IV MDD (recurrent, mod-severe)

Outcome	Study	Subcohort	Definitions
		i2b2-TRD 13	ICD-9 MDD, EMR review, SSRI-responsive or unresponsive to ≥2 AD trials
		Janssen 14,15	DSM-IV MDD (recurrent)
		MARS 16-18	DSM-IV MDD
		NESDA/NTR: NESDA 19,20	DSM-IV MDD
		NESDA/NTR: NTR 19,20	DSM-IV MDD
		Pfizer 9	DSM-IV MDD
		QIMR 13,21	DSM-IV MDD
		RADIANT-UK 22	DSM-IV MDD (recurrent in DeCC & DeNT; MDD FHx in DeNT)
		RADIANT-GER 22	DSM-IV MDD (recurrent in DeCC & DeNT; MDD FHx in DeNT)
		RADIANT-IRISH 22,23	DSM-IV MDD (recurrent in DeCC & DeNT; MDD FHx in DeNT)
		RADIANT-US 22,24	DSM-IV MDD (recurrent in DeCC & DeNT; MDD FHx in DeNT)
		RADIANT-DEN 22	DSM-IV MDD (recurrent in DeCC & DeNT; MDD FHx in DeNT)
		Roche †	DSM-IV MDD; MADRS≥25; non-response to 1-3 ADs
		Rotterdam 25	DSM-IV MDD
		SHIP 0 26	DSM-IV MDD
		SHIP-TREND 26	DSM-IV MDD
		STAR*D 27	DSM-IV MDD
		TwinGene 28,29	Inpatient MDD tx; outpatient MDD tx & ≥1 AD Rx; CIDI MDD & 1 AD Rx; CIDI recurrent MDD
		UK Biobank	Broad depression was defined by the participants' response to the questions 'Have you ever seen a general practitioner for nerves, anxiety, tension or depression?' or 'Have you ever seen a psychiatrist for nerves, anxiety, tension or depression?'. Exclusions were applied to participants who were identified with bipolar disorder, schizophrenia, or personality disorder using self-declared data following the approach of Smith, et al (PMID: 24282498)
	FG5	-	ICD9: 2961, 2968, 3004. ICD10: F3[2-3].
Schizophrenia	UKB	-	ICD9: 295. ICD10: F20, F25. Self-report (field 20002): 1289.
	PGS	-	Individuals with schizophrenia or schizoaffective disorder were included as cases. Details on all individual studies available in the Supplementary Methods (pages 2-12). PMID: 25056061.
	FG5	-	ICD8: 295|297|298[1-9]|299. ICD9: 295|29[7-8]. ICD10: F2.

T2D, type 2 diabetes. DIA, DIAGRAM Consortium; FG5, FinnGen Freeze 5 consortium; UKB, UK Biobank; HOW, Howard 2019; PGC, Psychiatric Genomics Consortium.

Table S2. Variant-level associations of the single-nucleotide polymorphisms used as instrumental variables for plasma cortisol and their associations with five main outcomes their corresponding source GWAS and in pooled consortia.

**Trait**		**Study**	**SNP: rs11621961**<br>**Gene: *SERPINA6***<br>**EA: C; OA: T**		**SNP: rs12589136**<br>**Gene: *SERPINA6***<br>**EA: T; OA: G**		**SNP: rs2749527**<br>**Gene: *SERPINA1***<br>**EA: C; OA: T**	
			**Beta (SE)**	***P***	**Beta (SE)**	***P***	**Beta (SE)**	***P***
Exposure	Plasma Cortisol	CORNET	0.08 (0.013)	4.0×10^{-8}	0.10 (0.015)	3.3×10^{-12}	0.08 (0.013)	5.2×10^{-11}
Main outcomes	T2D	DIA	1.60×10^{-3} (0.007)	0.81	7.50×10^{-3} (0.008)	0.33	6.50×10^{-3} (0.006)	0.31
		FG5	2.00×10^{-3} (0.011)	0.86	2.06×10^{-2} (0.013)	0.12	6.80×10^{-3} (0.011)	0.53
		DIA, FG5	1.70×10^{-3} (0.006)	0.77	1.08×10^{-2} (0.007)	0.11	6.58×10^{-3} (0.005)	0.23
	Hypertension	UKB	1.22×10^{-2} (0.006)	0.03	3.27×10^{-3} (0.007)	0.62	5.14×10^{-3} (0.005)	0.35
		FG5	1.30×10^{-2} (0.010)	0.17	1.26×10^{-2} (0.011)	0.26	1.05×10^{-2} (0.009)	0.25
		UKB, FG5	1.24×10^{-2} (0.005)	0.01	5.72×10^{-3} (0.006)	0.32	6.56×10^{-3} (0.005)	0.16
	Osteoporosis	UKB	9.35×10^{-3} (0.018)	0.61	1.27×10^{-3} (0.022)	0.95	-1.20×10^{-2} (0.018)	0.50
		FG5	4.19×10^{-2} (0.027)	0.12	9.50×10^{-3} (0.032)	0.77	-1.99×10^{-2} (0.026)	0.44
		UKB, FG5	1.96×10^{-2} (0.015)	0.20	3.90×10^{-3} (0.018)	0.83	-1.45×10^{-2} (0.015)	0.32
	Depression	FG5	-2.00×10^{-4} (0.012)	0.99	2.03×10^{-2} (0.013)	0.13	1.19×10^{-2} (0.011)	0.27
		HOW	1.50×10^{-3} (0.005)	0.74	-3.70×10^{-3} (0.005)	0.49	-3.9×10^{-3} (0.004)	0.36
		HOW, FG5	1.27×10^{-3} (0.004)	0.76	-4.53×10^{-4} (0.005)	0.93	-1.77×10^{-3} (0.004)	0.66
	Schizophrenia	UKB	2.93×10^{-3} (0.073)	0.97	5.58×10^{-4} (0.086)	0.99	-1.28×10^{-2} (0.071)	0.86
		PGC	9.00×10^{-4} (0.011)	0.94	-2.50×10^{-3} (0.013)	0.85	1.37×10^{-2} (0.011)	0.19
		FG5	-1.79×10^{-2} (0.023)	0.44	-8.40×10^{-3} (0.027)	0.76	-4.66×10^{-2} (0.022)	0.03
		UKB, PGC, FG5	-2.67×10^{-3} (0.010)	0.79	-3.52×10^{-3} (0.011)	0.76	1.98×10^{-3} (0.009)	0.83
Related traits	SBP	UKB	3.90×10^{-3} (0.002)	0.11	8.78×10^{-4} (0.003)	0.76	1.28×10^{-3} (0.002)	0.58
		Evangelou 2018	3.26×10^{-3} (0.002)	0.10	2.25×10^{-3} (0.002)	0.84	2.08×10^{-3} (0.002)	0.16
	DBP	UKB	4.19×10^{-3} (0.002)	0.08	-2.48×10^{-3} (0.003)	0.39	1.63×10^{-3} (0.002)	0.48
		Evangelou 2018	2.71×10^{-3} (0.002)	0.10	3.69×10^{-4} (0.002)	0.85	2.16×10^{-3} (0.001)	0.17

EA, effect allele; OA, other allele; SE, standard error; SNP, single-nucleotide polymorphism; CORNET, CORtisol NETwork consortium. DIA, DIAGRAM Consortium; UKB, UK Biobank. FG5, FinnGen Freeze 5 consortium. PGC, Psychiatric Genomics Consortium; HOW, Howard 2019[2]; T2D, type 2 diabetes; SBP, systolic blood pressure; DBP, diastolic blood pressure.

Table S3. Power calculation for separate studies and pooled meta-analyses for main binary outcomes. The significance level is set at 0.05, the true OR approximated from Hackett et al. 2016 is 1.18, and the proportion of variation in cortisol explained by SNPs is 0.0054 (Bolton et al. 2014). K represents proportion of cases. Power calculation for continuous outcomes is omitted due to lack of study with comparable populations and cortisol assessment method to reliably approximate the true effects of cortisol on SBP and DBP.

**Outcome**	**Study**	**Total**	**K**	**Power**
				**OR=1.18**
T2D	DIA	898,130	0.083	92.5
	FG5	211,766	0.138	53.6
	DIA, FG5	1,109,896	0.093	97.9
Hypertension	UKB	367,542	0.284	93.2
	FG5	218,754	0.256	73.5
	UKB, FG5	586,296	0.274	99.1
Osteoporosis	UKB	367,542	0.018	18.4
	FG5	212,778	0.015	11.5
	UKB, FG5	580,320	0.017	25.2
Depression	FG5	215,644	0.109	46.7
	HOW	500,199	0.341	98.7
	HOW, FG5	715,843	0.271	99.7
Schizophrenia	UKB	367,542	0.001	5.8
	PGC	82,315	0.431	41.7
	FG5	218,792	0.046	25.1
	UKB, PGC, FG5	668,649	0.069	77.2

UKB, UK Biobank; FG5, FinnGen Freeze 5 consortium; PGC, Pyschiatric Genomics Consortium; HOW, Howard 2019; T2D, type 2 diabetes.

Table S4. Correlation matrix of the three single-nucleotide polymorphisms in the 1000 Genomes Project, European-ancestry subpopulation. This matrix was used to account for SNP correlation in all univariable and multivariable MR analyses.

SNP	rs11621961	rs12589136	rs2749527
rs11621961	1	-0.233301	-0.437229
rs12589136	-0.233301	1	0.491219
rs2749527	-0.437229	0.491219	1

SNP, single-nucleotide polymorphism

Figure S3. Beta-beta scatter plots of exposure-outcome associations generated from the largest dataset for each outcome.

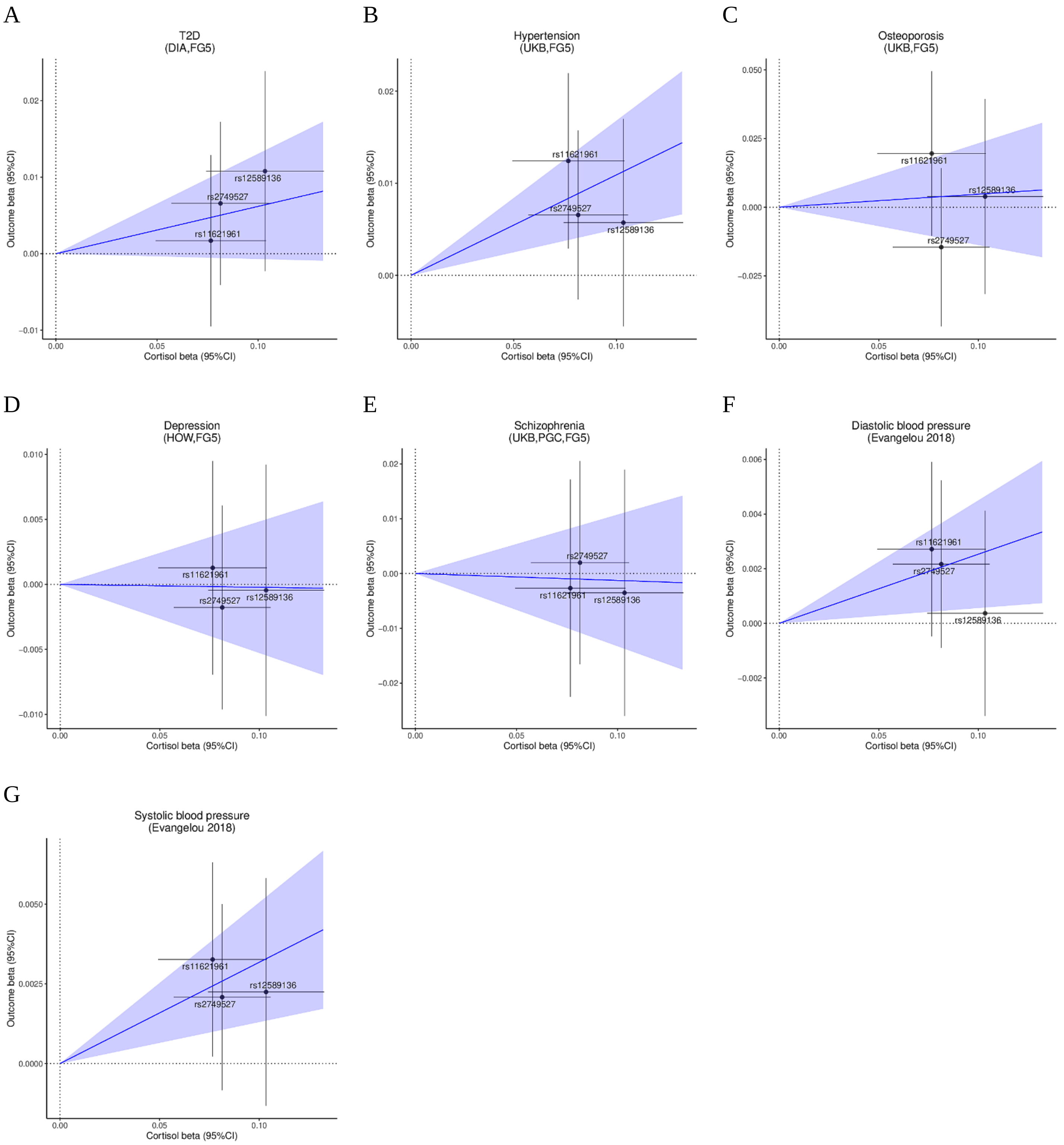

T2D, type 2 diabetes; DIA, DIAGRAM Consortium; FG5, FinnGen Freeze 5 consortium; UKB, UK Biobank; HOW, Howard 2019; PGC, Pyschiatric Genomics Consortium.

Supplemental references

1. Lawlor DA, Harbord RM, Sterne JA, Timpson N, Davey Smith G. Mendelian randomization: using genes as instruments for making causal inferences in epidemiology. *Stat Med.* 2008;27(8):1133-1163.
2. Howard DM, Adams MJ, Clarke TK, et al. Genome-wide meta-analysis of depression identifies 102 independent variants and highlights the importance of the prefrontal brain regions. *Nat Neurosci.* 2019;22(3):343-352.
